# Histological architectural classification determines recurrence pattern and prognosis after curative hepatectomy in patients with hepatocellular carcinoma

**DOI:** 10.1371/journal.pone.0203856

**Published:** 2018-09-14

**Authors:** Hirohisa Okabe, Tomoharu Yoshizumi, Yo-ichi Yamashita, Katsunori Imai, Hiromitsu Hayashi, Shigeki Nakagawa, Shinji Itoh, Norifumi Harimoto, Toru Ikegami, Hideaki Uchiyama, Toru Beppu, Shinichi Aishima, Ken Shirabe, Hideo Baba, Yoshihiko Maehara

**Affiliations:** 1 Department of Gastroenterological Surgery, Graduate School of Life Sciences, Kumamoto University, Kumamoto, Japan; 2 Department of Surgery and Science, Graduate School of Medical Sciences, Kyushu University, Fukuoka, Japan; 3 Department of Multidisciplinary Treatment for Gastroenterological Cancer, Kumamoto University Hospital, Kumamoto, Japan; 4 Department of Surgery, Yamaga Central Medical Center, Kumamoto, Japan; 5 Department of Pathology, Saga University Hospital, Saga, Japan; 6 Department of Thoracic and Visceral Organ Surgery, Gunma University Graduate School of Medicine, Gunma, Japan; The University of Tokyo, JAPAN

## Abstract

**Aim:**

The clinical impact of pathological classification based on architectural pattern in hepatocellular carcinoma (HCC) remains elusive in spite of its well-known and common feature.

**Methods:**

The prognostic impact of pathological classification was examined with prospective database. Three hundred and eighty HCC patients who underwent curative hepatectomy as an initial treatment in Kumamoto University were enrolled as a test cohort. The outcome was confirmed with a validation cohort in Kyushu University.

**Results:**

Macrotrabecular (macro-T) subtype (n = 38) and compact subtype (n = 43) showed similar biological and prognostic features. Both showed higher AFP level and worse overall survival than microrabecular (micro-T) subtype (n = 266). Multivariate analysis for overall survival revealed that DCP ≥ 40, multiple tumor and macro-T/compact subtype were associated with poor overall survival (risk ratio = 2.2, 1.6 and 1.6; p = 0.002, 0.020, and 0.047, respectively). Of note, 32% of macro-T/compact subtype showed early recurrence within 1 year, which showed substantially low (5%) 5 year overall survival, whereas 16% of micro-T/PG subtype did. Twenty-one percent of macro-T/compact subtype showed multiple intrahepatic metastases (≥ 4) or distant metastases, which resulted in non-curative treatment, whereas 5% of micro-T/PG subtype did. In validation cohort, macro-T/compact subtype was an independent predictor of worse overall survival.

**Conclusion:**

Macro-T/compact subtype is biologically discriminated from micro-T and PG subtypes due to its aggressive features and poor prognosis after curative treatment. Additional treatment with curative hepatectomy on Macro-T/compact subtype should be discussed because of high possibility of systemic residual cancer cell.

## Introduction

Hepatocellular carcinoma (HCC) is the sixth most common malignant tumors and the third most common cause of death in the world [1]. We have to address the high frequency of recurrence after curative surgery and chose the appropriate treatment. There are unsolved issues that are 1) limited choice of effective chemotherapeutic agent and 2) unestablished additional treatment on surgery such as neoadjuvant therapy or adjuvant therapy. Sorafenib and regorafenib are accepted chemotherapeutic agent for improving the patient survival, however the efficacy is still limited [2, 3].

As shown in pathological classification of HCC[4], there are three types of pathological classifications in HCC; architectural pattern, cytological variants and grading. Although all classifications are important for pathologically understanding the tumor biology, clinical impact of them is not clearly determined. Architectural pattern is based on trabeculae composed by hepatocytes which are well-known histological structure in the normal liver and are surrounded by sinusoidal space where blood stream flows from portal vein to central vein. Hepatocytes produce bile acid and secrete it in biliary canaliculae which face opposite side to sinusoidal space. In HCC cells, trabeculae become thicker and thick trabeculae is considered to be de-defferentiated HCC cells[4]. Previous investigation suggested that compact and thick trabecular subtypes are reported to show higher malignant potential than other subtype[5]. However, detailed clinical course based on architectural classification remains unclear.

We found the robust impact of architectural classification on recurrence pattern and prognosis after curative surgery in patients with HCC. Recurrence pattern was highly implicated in treatment strategy. Prognostication with architectural classification was confirmed by test cohort in our institution and validation cohort in the other institution.

## Patients and methods

### Patients

In the test cohort, from April 2005 to August 2010, total number of hepatectomy on HCC patients in the department of gastroenterological surgery, Kumamoto University was 651. Among them, consecutive 390 patients with HCC who underwent curative hepatic resection as an initial treatment were evaluated. Patients were pathologically diagnosed as HCC. Pathological findings including an architectural pattern were routinely obtained by experienced pathologists for determining the final stage of the tumor. Trabecular pattern was determined by the number of cords of tumour cells separated by sinusoidal-like vascular spaces. In macrotrabecular pattern, cords were thicker than 5–7 cells, whereas cords were composed of mostly 2–4 cells in microtrabecular pattern. The compact pattern was characterized by extensive compacted hepatocytes without sinusoidal space, resulting in its solid appearance. Patients with combined HCC and cholangiocarcinoma were excluded. In the validation cohort, consecutive 180 patients with HCC who underwent curative hepatic resection in the department of surgery and science, Kyushu University were analyzed. The patients signed informed consent for the current clinical study. Both Kumamoto University and Kyushu University Institutional review boards approved the study.

### Statistical analysis

Median value was used as cut-off value for prognostic analysis. Variables with a normal distribution were expressed as mean ± standard deviation (SD); statistical comparisons between the measures or groups were performed using the Wilcoxon test and analysis of variance (ANOVA). Measures with a discrete distribution were expressed as counts (%) and analyzed by the χ2 test or Fischer’s exact test. For overall survival (OS) and relapse-free survival (RFS), we used the log-rank test and the Kaplan-Meier method. Univariate and multivariate Cox-proportional hazard analysis was performed to estimate the HR of prognostic factors. The Harrell c-index was used in the prognostic models [6]. Predictive accuracy was evaluated by the c-index, which ranges from 0.5 (no predictive ability) - 1.0 (perfect discrimination). P values less than 0.05 were considered significant. Statistical analyses were performed using the JMP program version 10 (SAS Institute, Cary, NC) and R 3.4.4.

## Results

### Clinical characteristics of patients based on histopathological classification

**Fig 1:**
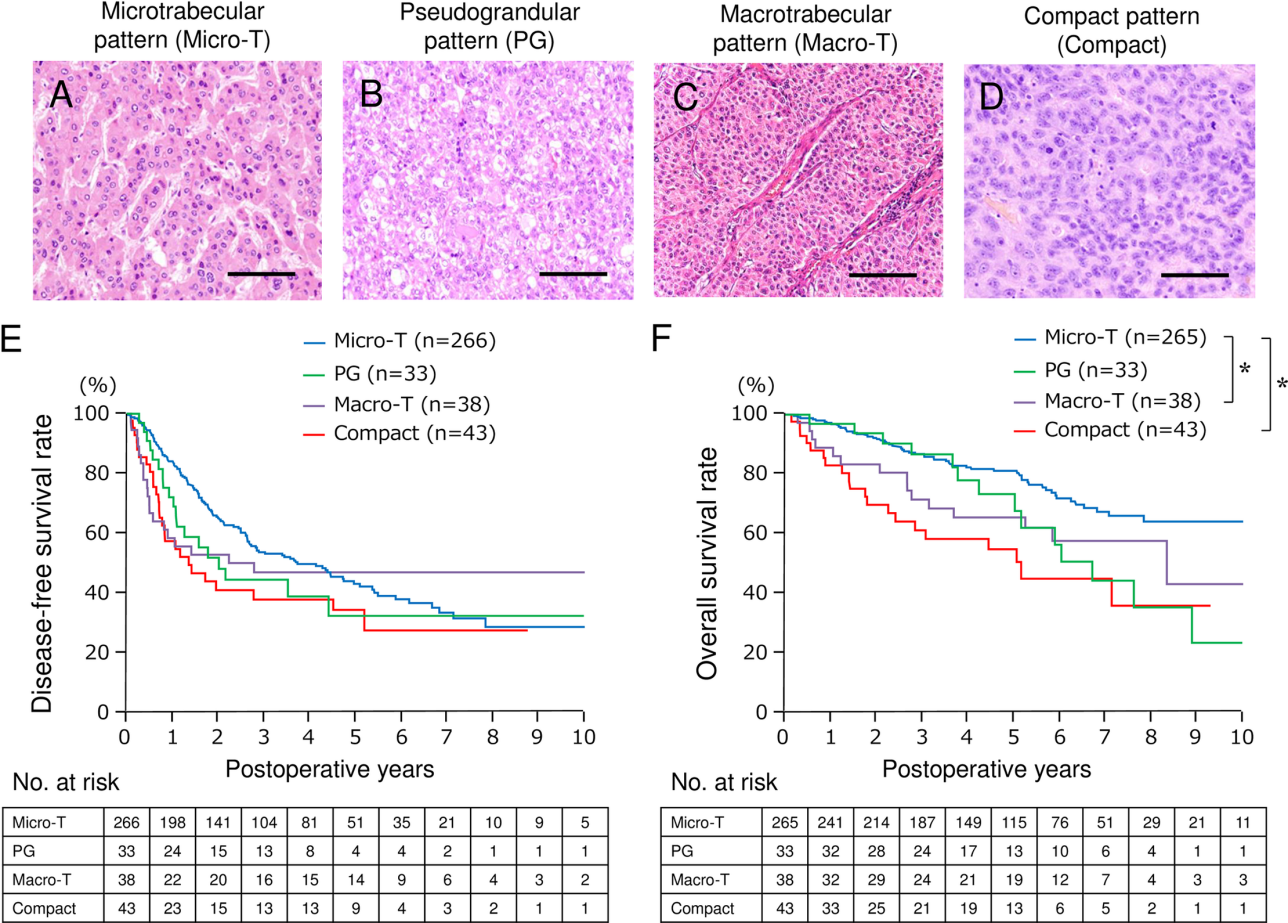
Postoperative outcome based on architectural classification. Representative HE pictures of four major subtypes are shown; Microtrabecular subtype (A), seudograndular subtype (B), Macrotrabecular subtype (C), and compact subtype (D). Disease-free survival (E) and overall survival (F) of each subtype is shown. Overall survival of Micro-T is significantly better than that of Macro-T (*p* = 0.0413) and Compact (*p* < .0001). Bars, 100 μm. Micro-T, microtrabecular subtype; PG, pseudoglandular subtype; Macro-T, macrotrabecular subtype; Compact, compact subtype. *, p < 0.05.

Based on pathological architectural classification, patients with HCC were classified into 4 sybtypes; microtrabecular subtype (Micro-T), Pseudoglandular subtype (PG), macrotrabecular subtype (Macro-T), and compact subtype (C) (Fig 1A–1D). Other subtypes including scirrhous subtype (< 0.1%) and sarcomatoid subtype (< 0.1%) were excluded from the analysis because of their exceptional features. Clinicopathological characteristics were compared among groups (Table 1). Biological feature regarding tumor progression of Macro-T subtype is quite similar to compact subtype rather than to Micro-T subtype. In order to confirm the prognostic impact of architectural classification, disease-free survival (Fig 1E) and overall survival (Fig 1F) was compared among four subtypes. Overall survival of Macro-T and compact subtype was worse than that of Micro-T subtype (p = 0.0413 and < .0001, respectively).

**Table 1:** Characteristics of patients based on pathological architectural features (n = 382).

	Micro-T (n = 266)	PG (n = 33)	Macro-T (n = 38)	Compact (n = 43)	P-value
Gender Male	206 (77%)	29 (88%)	26 (68%)	35 (81%)	0.2378
Age† (years)	68	68	64.5	67	0.3453
AFP† (U/mL)	11.1	12.2	651.5	52.9	< .0001
PIVKAII† (U/mL)	62	188	600	200	0.0009
Triple positive	39 (15%)	1 (3%)	16 (42%)	18 (42%)	< .0001
ICG R15† (%)	11.8	12.2	9.2	12.0	0.0921
HCV-Ab (+)	127 (48%)	14 (44%)	16 (43%)	24 (55%)	0.6553
Albumin† (g/dl)	4.0	4.0	4.1	3.9	0.3643
Tumor size† (mm)	32	34.5	52.5	40	0.0071
Tumor number (solitary)	183 (72%)	25 (78%)	21 (57%)	23 (56%)	0.0388
Vascular invasion (+)	81 (31%)	10 (31%)	25 (66%)	26 (60%)	< .0001
Gross morphology SN	183 (72%)	25 (78%)	21 (57%)	23 (56%)	0.0388
Liver cirrhosis‡	98 (37%)	7 (21%)	13 (34%)	22 (51%)	0.0614

Micro-T, microtrabecular; PG, Pseudoglandular; macro-T, macrotrabecular; SN, simple nodular, AFP; Alpha-fetoprotein, PIVKA-II; protein induced by Vitamin K absence or antagonists-II

† Median value

‡ Defined by F4 stage from new Inuyama classification (Ichida et al. Int Hepatol Commun 1996)

### Clinical feature and prognostic impact of macro-T/C subtype

**Fig 2:**
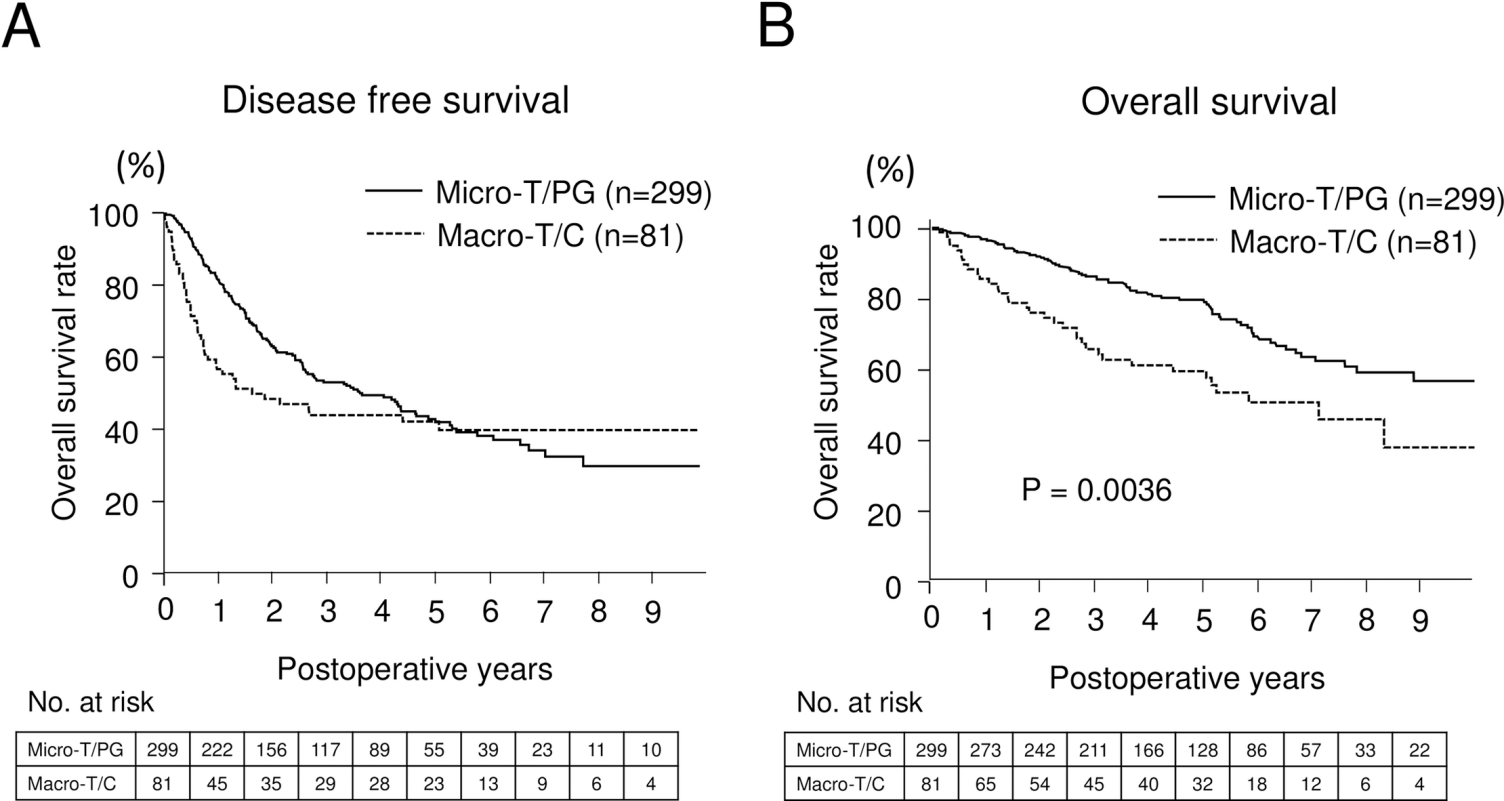
Postoperative outcome comparing Micro-T/PG subtype versus Macro-T/C subtype in test set. Disease-free survival (A) and overall survival (B) of Micro-T/PG subtype versus Macro-T/C subtype is shown. Three hundred and eighty patients in Kumamoto University were analyzed as test set.

Given the biologically similar feature of macro-T and compact subtype, clinical and biological characteristics of these subtypes (Macro-T/C) were compared to that of others (Table 2). Macro-T/C subtype has a robust correlation to clinical parameters relevant to tumor progression, but not to background liver status. Univariate analysis for overall survival revealed that tumor abnormal des-c-carboxy prothrombin (DCP) level, triple positive tumor markers, alpha-fetoprotein (AFP), lens culinaris agglutinin-reactive fraction of AFP (AFP-L3), and DCP, defined by Kiriyama S et al. [7], large tumor size, multiple tumor number, vascular invasion positive, gross morphology excepting simple nodular subtype, and Macro-T/C subtype were associated with poor overall survival. Multivariate analysis for overall survival revealed that DCP ≥ 40, multiple tumor number and Macro-T/C subtype were independent factors associated with poor survival (Table 3). We did not show differentiation status which was highly correlated with architectural classification, because both tumor grading and architectural classification were biologically very similar and relevant. We also administered tumor grading instead of architectural classification and performed same prognostic analysis on this cohort. We found that poorly differentiated tumor was associated with poor OS, but it was not independent factor associated with poor survival (data not shown). On the basis of the current classification, the HRs were 2.16 (1.316–3.661, P = 0.0020) for DCP ≥ 40, 1.63 (1.082–2.411, P = 0.0195) for multiple tumor, and 1.56 (1.006–2.389, P = 0.0472) for Macro-T/C subtype. The c-index of the multivariable prediction of overall survival, including DCP, tumor number, and Macro-T/C subtype, was 0.644. Disease-free survival (Fig 2A) and overall survival (Fig 2B) of Macro-T/C subtype versus Micro-T/PG subtype are shown in Fig 2.

**Table 2:** Characteristics of patients based on pathological architectural subgroups (n = 382).

	Micro-T/PG (n = 299)	Macro-T/C (n = 81)	P-value
Gender Male	235 (79%)	61 (75%)	0.5272
Age† (years)	68	65	0.0695
AFP† (U/mL)	11.1	125.2	< .0001
DCP† (U/mL)	68	279	0.0003
Triple positive	40 (13%)	34 (42%)	< .0001
ICG R15† (%)	11.8	10.6	0.0878
HCV-Ab (+)	141 (48%)	40 (50%)	0.7461
Albumin† (g/dl)	4.0	4.0	0.6944
Tumor size† (mm)	32	45	0.0009
Tumor number (solitary)	221 (75%)	50 (62%)	0.0218
Vascular invasion (+)	91 (31%)	51 (63%)	< .0001
Gross morphology SN	208 (73%)	44 (56%)	0.0049
Liver cirrhosis‡	105 (35%)	35 (43%)	0.1943

Micro-T, microtrabecular; PG, Pseudoglandular; macro-T, macrotrabecular; AFP; Alpha-fetoprotein, des-c-carboxy prothrombin, DCP; SN, simple nodular,

† Median value

‡ Defined by F4 stage from new Inuyama classification (Ichida et al. *Int Hepatol Commun* 1996)

**Table 3:** Univariate and multivariate analysis for overall survival in test set (n = 382).

			Univariate analysis		Multivariate analysis		
		n	MST (Mo)	P-value	Hazard Ratio	95% CI	P-value
AFP† (U/mL)	≥ 20	165	53.3	0.1029			
	< 20	217	57.6				
DCP (U/mL)	≥ 40	241	49.0	< .0001	2.16	1.316–3.661	0.0020
	< 40	141	60.8				
Triple positive^†^	Positive	74	34.0	0.0082	1.09	0.677–1.783	0.7378
	Negative	308	60.2				
Tumor size* (mm)	≥ 34	190	46.7	0.0001	1.49	0.985–0.9999	0.0592
	< 34	188	45.3				
Tumor number	Multiple	106	49.1	0.0064	1.63	1.082–2.411	0.0195
	Solitary	273	57.9				
Vascular invasion	Positive	143	46.7	0.0005	1.39	0.933–2.073	0.1053
	Negative	233	60.7				
Gross morphology	SN	254	57.7	0.0109	0.80	0.534–1.213	0.2903
	Others	111	42.7				
Architectural subtype	Macro-T/C	81	38.1	0.0002	1.56	1.006–2.389	0.0472
	Micro-T/PG	299	58.0				

Micro-T, microtrabecular; PG, Pseudoglandular; Macro-T, macrotrabecular; SN, simple nodular, des-c-carboxy prothrombin; DCP

^†^ Abnormal level of three HCC markers; alpha-fetoprotein (AFP), lens culinaris agglutinin-reactive fraction of AFP (AFP-L3), and DCP (Kiriyama S et al. *Ann Surg* 2011)

*Cut-off value was determined by median value.

### Relevance of pathological classification to recurrence pattern

**Fig 3:**
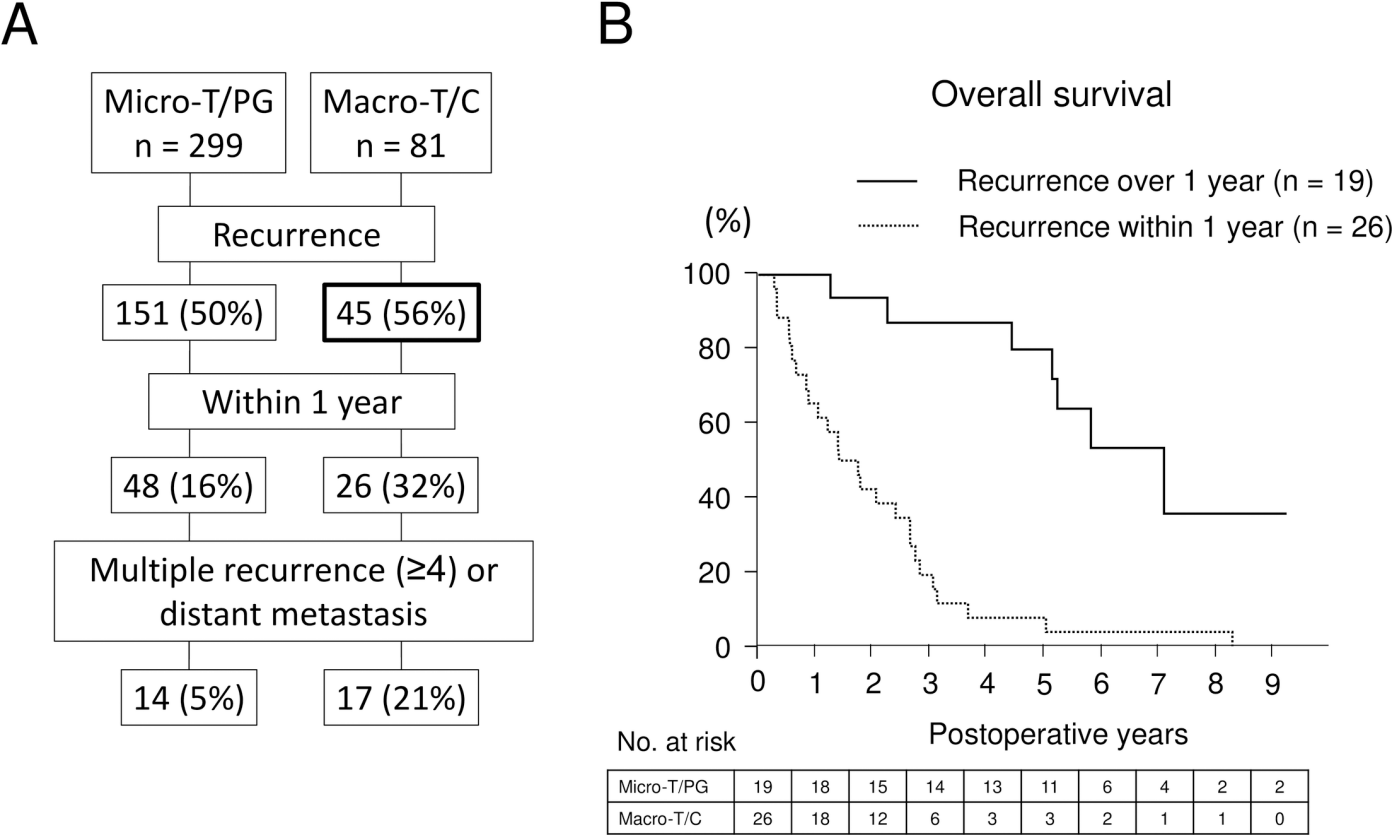
Recurrence pattern based on pathological subtypes. A. Recurrence patterns of Micro-T/PG subtype versus Macro-T/C subtype after curative surgery are shown. B. Among 45 patients who showed recurrence after curative surgery in Macro-C subtypes, 26 patients (32% of all) showed the recurrence within 1 year leading to extremely poor 5-year survival.

Since early death within 2 years, which is considerably caused by intrahepatic metastasis of primary tumor [8], is remarkable, postoperative course and recurrence pattern was carefully examined (Table 4 and Fig 3A). Although the occurrence of intrahepatic metastases was higher in Micro-T/PG subtype than Macro-T/C subtype, Macro-T/C subtype showed higher number of early recurrence within 1 year (p = 0.0026) and intrahepatic metastases (p = 0.0337) at the recurrence resulting in non-curative treatment such as systemic chemotherapy and trans-arterial chemoembolization (TACE) (p = 0.0062). On the other hand, the number of patients who could receive hepatectomy or RFA was higher in patients with Micro-T/PG subtype than those with Macro-T/C subtype (p = 0.0025). Macro-T/C subtype showed higher occurrence of distant metastasis (p = 0.0387). Of note, early recurrence within 1 year occurs in Macro-T/C subtype more highly than Micro-T/PG subtype does (32% vs 16%; Fig 3A and Table 4), leading to miserable 5-year overall survival up to 5% as compared to Macro-T/C subtype with recurrence occurring over 1 year (Fig 3B). In patients with early recurrence within 1 year (n = 74), either multiple recurrence (≥4) or distant metastasis were significantly more seen in Macro-T/C subtype (n = 17) than Micro-T/PG (n = 14) (p < 0001; Fig 3A).

**Table 4:** Recurrence pattern (n = 196).

	Micro-T/PG (n = 151)	Macro-T/C (n = 45))	P-value
*Intrahepatic recurrence*			
Total	142 (94%)	37 (82%)	0.030
Early recurrence within 1 year	48 (16%)	26 (32%)	0.026
Tumor number ≥ 4	26 (17%)	15 (33%)	0.0337
*Distant metastasis*			
Total	14 (9%)	10 (22%)	0.0387
Lung	6 (4%)	8 (18%)	0.0047
Bone	3 (2%)	0	0.7940
Lymph node	3 (2%)	2 (4%)	0.7045
Brain	2 (1%)	0	0.4848
*Treatment on recurrence*			
Hepatectomy/RFA	65 (43%)	8 (18%)	0.0025
TACE/HAI/Systemic chemotherapy	64 (42%)	30 (67%)	0.0062
Radiotherapy	0	1 (2%)	0.4098
Liver transplantation	1 (1%)	0	0.5191
BSC	4 (3%)	2 (4%)	0.6222

Micro-T, microtrabecular; PG, pseudoglandular; macro-T, macrotrabecular

We further examined whether pathological feature of recurrence is determined by that of primary tumor. Hepatectomy on recurrent lesion was performed in 26 patients. Twenty (87%) out of 23 patients with Micro-T/PG subtype showed same feature as primary tumor, whereas three (13%) out of them showed Macro-T/C subtype. All three patients with Macro-T/C subtype showed same feature as primary tumor. Five patients underwent 3^rd^ hepatectomy on re-recurence. All five patients showed same pathological features as primary recurrence (S1 Fig). Collectively, pathological feature of intrahepatic metastasis at recurrence is mostly determined by primary tumor except for a few cases with dedifferentiation of tumor.

### Subgroup analysis and clinical implication

In order to further understand the role of architectural classification in tumor development, subgroup analysis on overall survival was performed. Since percentage of Macro-T/C increases as tumor progresses (Figure A in S2 Fig), clinical impact of Macro-T/C subtype was considered to be important in late stage of HCC development. As we expected, in patients with stage I/II, overall survival of Macro-T/C subtype was comparable to that of Micro-T/PG (Figure B in S2 Fig). On the other hand, in patients with stage III/IVA, overall survival of Macro-T/C subtype was significantly worse than that of Micro-T/PG (p = 0.0046) (Figure C in S2 Fig). Similarly, in patients within Milan criteria, overall survival of Macro-T/C subtype was comparable to that of Micro-T/PG (Figure D in S2 Fig). Whereas, in patients out of Milan criteria, overall survival of Macro-T/C subtype was significantly worse than that of Micro-T/PG (p = 0.0041) (Figure E in S2 Fig). Taken together, Macro-T/C subtype is highly relevant to tumor development in advanced stage of HCC.

### Validation of prognostic impact of architectural classification

The prognostic impact of architectural classification was validated with another data set in Kyushu University. Consecutive 179 patients who underwent curative hepatectomy from 2004 to 2010 were enrolled. Macro-T/C subtype was associated with both poor disease-free survival (p = 0.0033) and overall survival (p = 0.0047) (S3 Fig). Multivariate analysis for overall survival revealed that only Macro-T/C subtype was an independent factor associated with poor overall survival after curative hepatectomy (S1 Table). The c-index of the prediction of overall survival including Macro-T/C subtype was 0.604.

## Discussion

Here we report the impact of pathological architectural pattern with special reference to recurrence pattern and clinical features in HCC. In this study, we intentionally selected patients who underwent hepatectomy as an initial treatment for HCC, because many patients with HCC undergo curative hepatectomy as a salvage surgery on recurrent tumor after non-curative treatment such as TACE and ablation therapy so that prognostic analysis might be biased by those patients. We found that Macro-T subtype is clinically similar to Compact subtype rather than Micro-T subtype. Macro-T/C subtype showed strong correlation to recurrence pattern and prognosis after curative surgery. Validation of the outcome in another institution confirmed that the Macro-T/C subtype has a robust impact on patient prognosis after curative surgery.

At present, it is well known that HCC is characterized by high possibility of intrahepatic recurrence after curative surgery. Early intrahepatic recurrence is considered to be intrahepatic metastasis originating from primary tumor resected at the surgery. Therefore, early recurrence within 2 years has a prognostic impact and clinical implication [8]. The current study revealed that recurrence pattern of Macro-T/C subtype is characterized by 1) early recurrence, 2) multiple intrahepatic recurrences, and 3) recurrences at distant organ especially in lung. Patients with Macro-T/C subtype exhibiting early recurrence within 1 year showed extremely poor survival. These facts suggest that Macro-T/C subtype has highly metastatic potential leading to systemic disease. Additionally, Macro-T/C subtype occurs in late stage of HCC, and prognostic impact of Macro-T/C subtype is significant only in late stage based on the subgroup analysis, suggesting that advanced HCC, but not early stage of HCC, with the pathological feature of Macro-T/C subtype does need additional treatment to control residual cancer cells.

Molecular phenotype of Macro-T/C subtype was introduced recently [9]. Macro-T/C subtype was associated with S2 subclass where AFP value was the highest, supporting the current outcome. High GPC3 and EpCAM expression were also appreciated in S2 subclass. EpCAM expression is associated with invasive phenotype and high histological grade in patients with HCC [10]. Mechanistically, blockade of β-Catenin signaling attenuates EpCAM+ population and proliferation of HCC cells [11]. Src-homology 2 domain–containing phosphatase 2 (Shp2), which has an role in the maintenance and self-renewal of embryonic and adult stem cells, promote the dedifferentiation of hepatoma cells via β-Catenin signaling. Shp2 highly expressed in EpCAM+ population also regulates tumorigenesis and chemosensitivity [12]. Another investigation revealed that Macro-T/C subtype was associated with cell cycle progression, cell proliferation, and activation of an oncogene *YAP* in HCC [13]. Interestingly, AFP by itself promotes metastasis of HCC cells by up-regulating metastasis-related proteins such as matrix petalloproteinase 2/9 and CXC chemokine receptor 4 [14]. Thus, translational study on therapeutically targetable molecules described above and relevant pathways in HCC patients is challenging but promising future subjects for us.

Since tumor presumably disseminates systemically at the time of hepatectomy for patients with macro-T/C subtype HCC, additional systemic treatment is necessary for them. Efficacy of Sorafenib is widely accepted for patients with unresectable advanced HCC but not for microscopic residual tumor after curative surgery [15, 16]. Recently, Regorafenib which is an oral multikinase inhibitor that blocks the activity of protein kinases involved in angiogenesis, oncogenesis, metastasis, and tumour immunity is also accepted for patients with unresectable HCC [3, 17]. Although the efficacy of Regorafenib as adjuvant chemotherapy is expected, mechanistic investigation for therapy is also important. HCC is well known as hypervascular tumor especially in advanced stage, however poorly differentiated tumor harbors anaerobic glucose metabolism detected by fluorodeoxyglucose positron emission tomography [18]. Clinical challenge targeting the mechanism depending on hypoxic condition is shown in phase III trial with Girentuximab, which is a major effector of the hypoxia inducible factor-1-mediated transcriptional response to tumor hypoxia and its critical role in tumor progression is well-recognized[19, 20], on high-risk renal cell carcinoma patients as adjuvant treatment following nephrectomy. Although the clinical benefit on OS and DFS were not demonstrated [21], on subset analysis, patients with high CA IX score experienced a statistically significant treatment effect with improved DFS (HR = 0.55; p = 0.01) [22]. Selecting patients with appropriate biomarker for additional treatment in addition to the curative surgery might be effective strategy for patients with HCC given the genetically diverse nature of solid tumor [23, 24].

We have to further address some limitations in the current study. Pathological architectural classification is based on the definition of WHO and was determined by a pathologist who is unaware of patient clinical information. We tried to make sure the definition of thick trabecular pattern by showing the number of cords of tumour cells. However, the detailed description discriminating thick trabecular and thin trabecular pattern is not provided in WHO definition, and thus we dare to describe it to emphasize the prognostic importance of macro-T/C subtype which prefers to lose sinusoidal space between hepatocytes and obtain a hypoxic condition. Although this issue needs consensus meeting with authorized liver pathologists, we here propose that thick trabecular subtype is clinically and biologically discriminated from thin trabecular subtype based on the current observational study.

## Conclusions

The current study is the first to show the clinical impact of histological architectural classification on recurrence pattern and prognosis after curative surgery in patients with HCC. It is revealed that Macro-T/C subtype is correlated with preoperative aggressive phenotype of tumor and results in higher early reccurence rate, higher number of liver metastasis, less chance of receiving hepatectomy/RFA on the recurrence, and poor prognosis after curative hepatectomy, especially for patients with advanced staged tumor. Postoperative clinical course is determined by histologic nature, and macro-T/C subtype with early recurrence needs additional treatment on top of the radical resection.

## Supporting information

S1 Table Univariate and multivariate analysis for overall survival in validation set (n = 179). (DOCX)

S1 Fig Histological recurrence pattern. Histological subtype of intrahepatic recurrent lesion is compared to that of primary tumor.
(TIF)

S2 Fig Overall survival based on subgroup analysis comparing Micro-T/PG subtype versus Macro-T/C subtype. A. Percentage of Macro-T subtype in each clinical stage is shown. B-E. Overall survival in patients with stage I/II (B), patients with stage III/IVA (C), patients within Milan (D), and patients out of Milan (E) is shown comparing Micro-T/PG subtype versus Macro-T/C subtype.
(TIF)

S3 Fig Postoperative outcome comparing Micro-T/PG subtype versus Macro-T/C subtype in validation set. Disease-free survival (A) and overall survival (B) of Micro-T/PG subtype versus Macro-T/C subtype is shown. One hundred and seventy-nine patients in Kyushu University were analyzed as validation set.
(TIF)

## Abbreviations:

HCC: hepatocellular carcinoma
AFP: alpha fetoprotein
DCP: des-c-carboxy prothrombin

## References

[1] Ferlay J, Shin HR, Bray F, Forman D, Mathers C, Parkin DM. Estimates of worldwide burden of cancer in 2008: GLOBOCAN 2008. Int J Cancer. 2010; 127:2893–2917. 10.1002/ijc.25516 21351269

[2] Cheng AL, Kang YK, Chen Z, Tsao CJ, Qin S, Kim JS, et al Efficacy and safety of sorafenib in patients in the Asia-Pacific region with advanced hepatocellular carcinoma: a phase III randomised, double-blind, placebo-controlled trial. Lancet Oncol. 2009; 10:25–34. 10.1016/S1470-2045(08)70285-7 19095497

[3] Bruix J, Qin S, Merle P, Granito A, Huang YH, Bodoky G, et al Regorafenib for patients with hepatocellular carcinoma who progressed on sorafenib treatment (RESORCE): a randomised, double-blind, placebo-controlled, phase 3 trial. Lancet. 2017; 389:56–66. 10.1016/S0140-6736(16)32453-9 27932229

[4] Bosman FT CF, Hruban RH, Theise ND. The International Agency for Research on Cancer. WHO Classification of Tumours of the Digestive System (IARC WHO Classification of Tumours). 4th ed.; 2010.

[5] Lauwers GY, Terris B, Balis UJ, Batts KP, Regimbeau JM, Chang Y, et al Prognostic histologic indicators of curatively resected hepatocellular carcinomas: a multi-institutional analysis of 425 patients with definition of a histologic prognostic index. Am J Surg Pathol. 2002; 26:25–34. 11756766 10.1097/00000478-200201000-00003

[6] Steyerberg EW, Eijkemans MJ, Harrell FE Jr., Habbema JD. Prognostic modeling with logistic regression analysis: in search of a sensible strategy in small data sets. Med Decision Making. 2001; 21:45–56. 10.1177/0272989X0102100106 11206946

[7] Kiriyama S, Uchiyama K, Ueno M, Ozawa S, Hayami S, Tani M, et al Triple positive tumor markers for hepatocellular carcinoma are useful predictors of poor survival. Ann Surg. 2011; 254:984–991. 10.1097/SLA.0b013e3182215016 21606837

[8] Portolani N, Coniglio A, Ghidoni S, Giovanelli M, Benetti A, Tiberio GA, et al Early and late recurrence after liver resection for hepatocellular carcinoma: prognostic and therapeutic implications. Ann Surg. 2006; 243:229–235. 10.1097/01.sla.0000197706.21803.a1 16432356 PMC1448919

[9] Tan PS, Nakagawa S, Goossens N, Venkatesh A, Huang T, Ward SC, et al Clinicopathological indices to predict hepatocellular carcinoma molecular classification. Liver Int. 2016; 36:108–118. 10.1111/liv.12889 26058462 PMC4674393

[10] Sung JJ, Noh SJ, Bae JS, Park HS, Jang KY, Chung MJ, et al Immunohistochemical Expression and Clinical Significance of Suggested Stem Cell Markers in Hepatocellular Carcinoma. J Pathol Transl Med. 2016; 50:52–57. 10.4132/jptm.2015.10.09 26581206 PMC4734967

[11] Yamashita T, Ji J, Budhu A, Forgues M, Yang W, Wang HY, et al EpCAM-positive hepatocellular carcinoma cells are tumor-initiating cells with stem/progenitor cell features. Gastroenterology. 2009; 136:1012–1024. 10.1053/j.gastro.2008.12.004 19150350 PMC2828822

[12] Xiang D, Cheng Z, Liu H, Wang X, Han T, Sun W, et al Shp2 promotes liver cancer stem cell expansion by augmenting beta-catenin signaling and predicts chemotherapeutic response of patients. Hepatology. 2017;65:1566–1580. 10.1002/hep.28919 28059452

[13] Perra A, Kowalik MA, Ghiso E, Ledda-Columbano GM, Di Tommaso L, Angioni MM, et al YAP activation is an early event and a potential therapeutic target in liver cancer development. J Hepatol. 2014; 61:1088–1096. 10.1016/j.jhep.2014.06.033 25010260

[14] Lu Y, Zhu M, Li W, Lin B, Dong X, Chen Y, et al Alpha fetoprotein plays a critical role in promoting metastasis of hepatocellular carcinoma cells. J Cell Mol Med. 2016; 20:549–558. 10.1111/jcmm.12745 26756858 PMC4759472

[15] Llovet JM, Ricci S, Mazzaferro V, Hilgard P, Gane E, Blanc JF, et al Sorafenib in advanced hepatocellular carcinoma. New Engl J Med. 2008; 359:378–390. 10.1056/NEJMoa0708857 18650514

[16] Bruix J, Takayama T, Mazzaferro V, Chau GY, Yang J, Kudo M, et al Adjuvant sorafenib for hepatocellular carcinoma after resection or ablation (STORM): a phase 3, randomised, double-blind, placebo-controlled trial. Lancet Oncol. 2015; 16:1344–1354. 10.1016/S1470-2045(15)00198-9 26361969

[17] Wilhelm SM, Dumas J, Adnane L, Lynch M, Carter CA, Schutz G, et al Regorafenib (BAY 73–4506): a new oral multikinase inhibitor of angiogenic, stromal and oncogenic receptor tyrosine kinases with potent preclinical antitumor activity. Int J Cancer. 2011; 129:245–255. 10.1002/ijc.25864 21170960

[18] Ijichi H, Shirabe K, Taketomi A, Yoshizumi T, Ikegami T, Mano Y, et al Clinical usefulness of (18) F-fluorodeoxyglucose positron emission tomography/computed tomography for patients with primary liver cancer with special reference to rare histological types, hepatocellular carcinoma with sarcomatous change and combined hepatocellular and cholangiocarcinoma. Hepatol Res. 2013; 43:481–487. 10.1111/j.1872-034X.2012.01107.x 23145869

[19] Pastorek J, Pastorekova S: Hypoxia-induced carbonic anhydrase IX as a target for cancer therapy: from biology to clinical use. Semin Cancer Biol. 2015; 31:52–64. 10.1016/j.semcancer.2014.08.002 25117006

[20] McDonald PC, Winum JY, Supuran CT, Dedhar S. Recent developments in targeting carbonic anhydrase IX for cancer therapeutics. Oncotarget. 2012; 3:84–97. doi: 10.18632/oncotarget.422 22289741 10.18632/oncotarget.422 PMC3292895

[21] Chamie K, Donin NM, Klopfer P, Bevan P, Fall B, Wilhelm O, et al Adjuvant Weekly Girentuximab Following Nephrectomy for High-Risk Renal Cell Carcinoma: The ARISER Randomized Clinical Trial. JAMA Oncol. 2017;3:913–920. 10.1001/jamaoncol.2016.4419 27787547 PMC5824229

[22] Arie S. Belldegrun KC, Pia Kloepfer, Barbara Fall, Paul Bevan, Stephan Störkel, et al ARISER: A randomized double blind phase III study to evaluate adjuvant cG250 treatment versus placebo in patients with high-risk ccRCC—Results and implications for adjuvant clinical trials. J Clin Oncol. 2013; 31:(suppl; abstr 4507).

[23] Schulze K, Nault JC, Villanueva A. Genetic profiling of hepatocellular carcinoma using next-generation sequencing. J Hepatol 2016; 65:1031–1042. 10.1016/j.jhep.2016.05.035 27262756

[24] Zucman-Rossi J, Villanueva A, Nault JC, Llovet JM. Genetic Landscape and Biomarkers of Hepatocellular Carcinoma. Gastroenterology. 2015; 149:1226–1239 e1224. 10.1053/j.gastro.2015.05.061 26099527

