# Supplementary material for: Histological architectural classification determines recurrence pattern and prognosis after curative hepatectomy in patients with hepatocellular carcinoma
Source: PLoS One. 2018 Sep 14;13(9):e0203856. doi: 10.1371/journal.pone.0203856 (PMC6138409; doi:10.1371/journal.pone.0203856)
Supplement: S1 Table — (DOCX) [file pone.0203856.s001.docx]

**S1 Table.** Univariate and multivariate analysis for overall survival in validation set (n = 179)

|  |  |  | Univariate analysis | | Multivariate analysis | | |
| --- | --- | --- | --- | --- | --- | --- | --- |
|  |  | n | MST (Mo) | P-value | Hazard Ratio | 95% CI | P-value |
| AFP† (U/mL) | ≥ 20 | 71 | 29.1 | 0.0516 | 1.53 | 0.76-3.17 | 0.2352 |
|  | < 20 | 106 | 37.8 |  |  |  |  |
| DCP (U/mL) | ≥ 71 | 86 | 39.4 | 0.8234 |  |  |  |
|  | < 71 | 88 | 40.2 |  |  |  |  |
| Tumor size* (mm) | ≥ 30 | 88 | 39.7 | 0.8314 |  |  |  |
|  | < 30 | 91 | 39.5 |  |  |  |  |
| Tumor number | Multiple | 14 | 60.5 | 0.8632 |  |  |  |
|  | Solitary | 165 | 39.5 |  |  |  |  |
| Vascular invasion | Positive | 55 | 39.7 | 0.1119 |  |  |  |
|  | Negative | 120 | 42.5 |  |  |  |  |
| Architectural subtype | Macro-T/C | 44 | 34.1 | 0.0047 | 2.16 | 1.07 – 4.33 | 0.0328 |
|  | Micro-T/PG | 135 | 40.3 |  |  |  |  |

Micro-T, microtrabecular; PG, pseudoglandular; macro-T, macrotrabecular; SN, simple nodular

‡ Defined by F4 stage from new Inuyama classification (Ichida et al. *Int Hepatol Commun* 1996)

*Cut-off was determined by median value
