# Supplementary figures and images for: Histological architectural classification determines recurrence pattern and prognosis after curative hepatectomy in patients with hepatocellular carcinoma
Source: PLoS One. 2018 Sep 14;13(9):e0203856. doi: 10.1371/journal.pone.0203856 (PMC6138409; doi:10.1371/journal.pone.0203856)

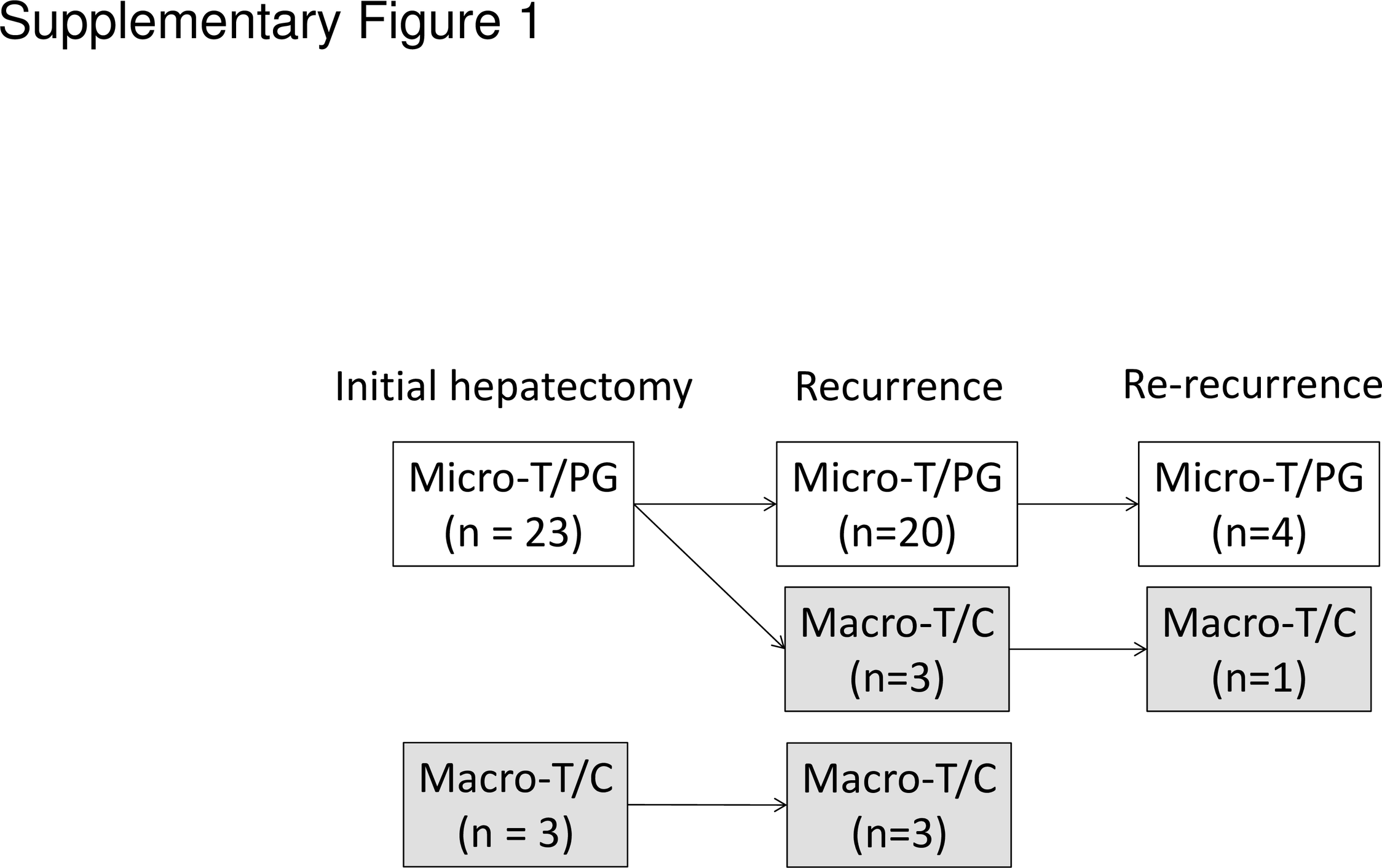

Supplement: S1 Fig — Histological subtype of intrahepatic recurrent lesion is compared to that of primary tumor. (TIF) [file pone.0203856.s002.tif]

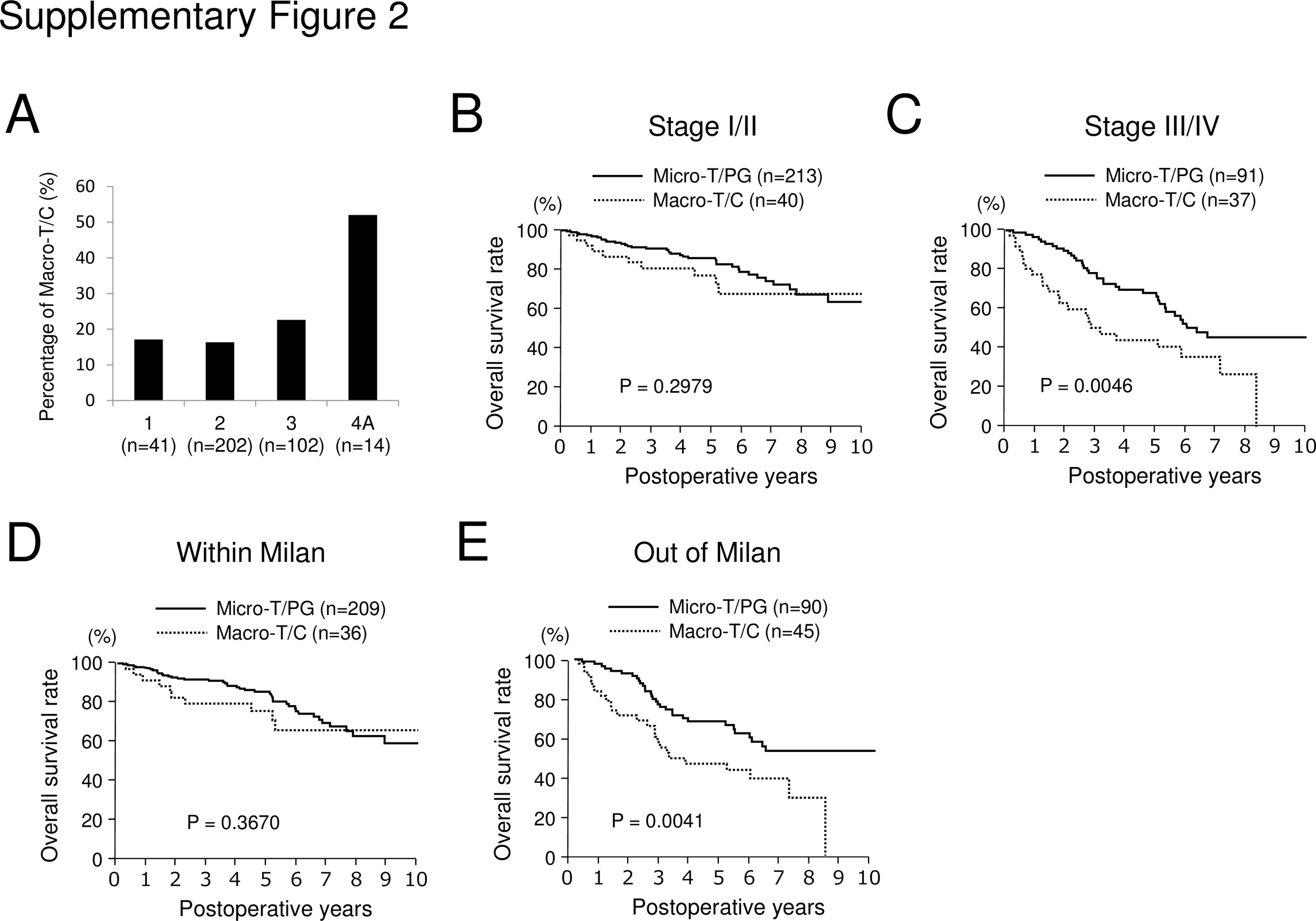

Supplement: S2 Fig — A. Percentage of Macro-T subtype in each clinical stage is shown. B-E. Overall survival in patients with stage I/II (B), patients with stage III/IVA (C), patients within Milan (D), and patients out of Milan (E) is shown comparing Micro-T/PG subtype versus Macro-T/C subtype. (TIF) [file pone.0203856.s003.tif]

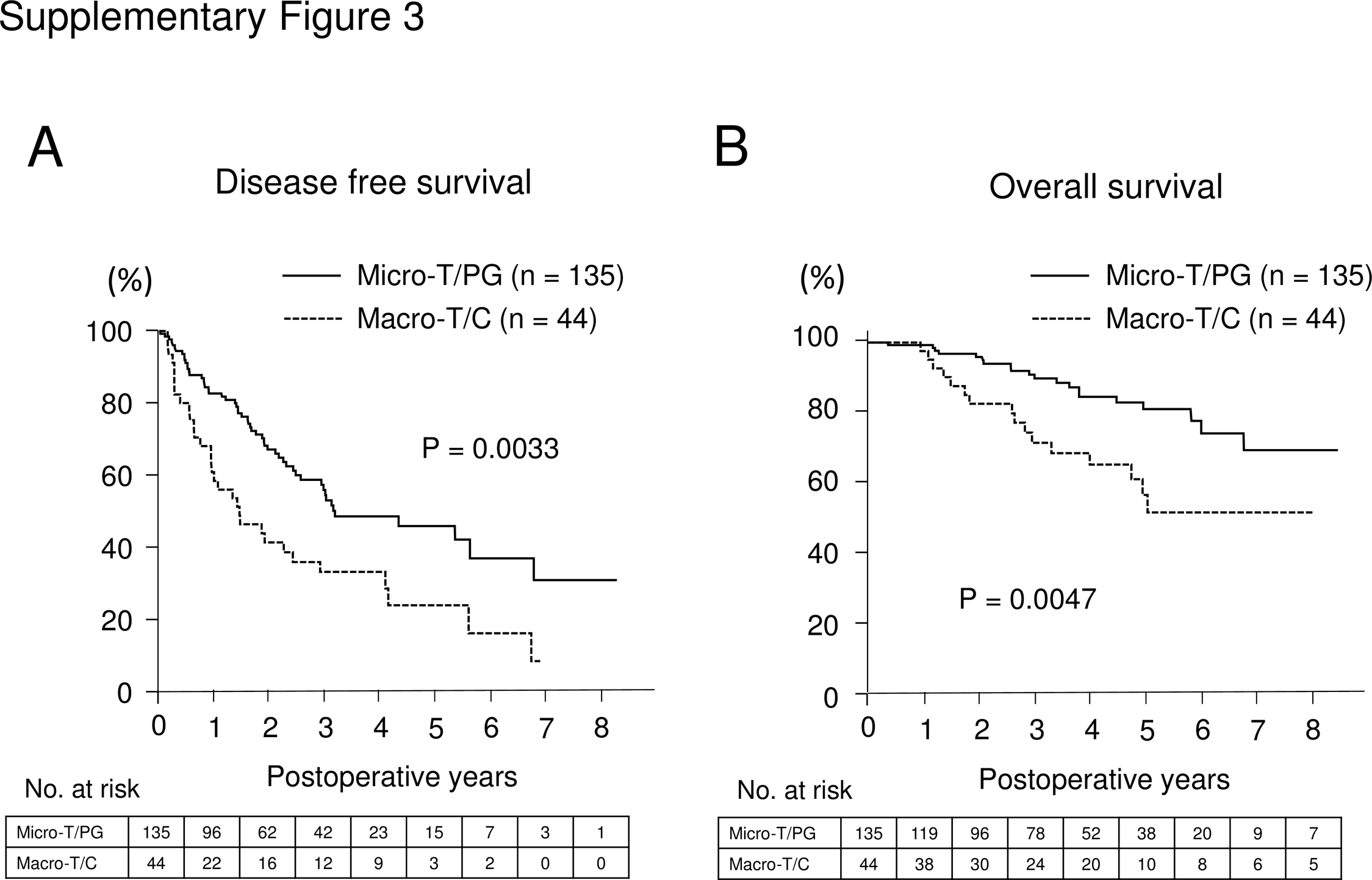

Supplement: S3 Fig — Disease-free survival (A) and overall survival (B) of Micro-T/PG subtype versus Macro-T/C subtype is shown. One hundred and seventy-nine patients in Kyushu University were analyzed as validation set. (TIF) [file pone.0203856.s004.tif]
